# Supplementary material for: VDAC1 negatively regulates melanogenesis through the Ca2+-calcineurin-CRTC1-MITF pathway
Source: Life Sci Alliance. 2022 Jun 1;5(10):e202101350. doi: 10.26508/lsa.202101350 (PMC9160443; doi:10.26508/lsa.202101350)
Supplement: Supplementary file 1 [file LSA-2021-01350_TableS1.docx]

**Supplementary Tables**

**Table S1.** siRNA sequences used in knockdown experiments.

| siRNA | sequences |
| --- | --- |
| Negative control (Gincel et al) | UUCUCCGAACGUGUCACGUTT |
| siRNA-*VDAC1*-human-1 | GUACGGCCUGACGUUUACATT |
| siRNA-*VDAC1*-human-4 | CTCCAGGTTAAAGTTGATTCA |
| siRNA-*Vdac1*-mouse-A | ACCCUGGGCACUGAGAUCATT |
