# Supplementary material for: VDAC1 negatively regulates melanogenesis through the Ca2+-calcineurin-CRTC1-MITF pathway
Source: Life Sci Alliance. 2022 Jun 1;5(10):e202101350. doi: 10.26508/lsa.202101350 (PMC9160443; doi:10.26508/lsa.202101350)
Supplement: Supplementary file 2 [file LSA-2021-01350_TableS2.docx]

**Supplementary Tables**

**Table S2.** Antibody details for protein detection.

| Protein | Secondary antibody | Supplier | Code |
| --- | --- | --- | --- |
| VDAC1 | rabbit pAb | Bioss | bs-1461R |
| Tyrosinase（M-19） | goat pAb | Santa Cruz | Sc-7834 |
| Tyrosinase Monoclonal Antibody（T311） | mouse mAb | Invitrogen | 35-6000 |
| anti-MITF（C5） | mouse mAb | Novusbio | NB110-10872 |
| TYRP1（G-9） | mouse mAb | Santa Cruz | Sc-166857 |
| anti-DCT | rabbit | Sigma | SAB4500626 |
| p-CREB（Ser133） | rabbit mAb | Cell Signaling | 9198 |
| TORC1/CRTC1（C71D11） | rabbit mAb | Cell Signaling | 2587 |
| β-actin | Mouse mAb | Sigma | A5441 |
| Histone H3.1 | rabbit pAb | Abmart | P30266l |
| GAPDH（D16H11） | rabbit mAb | Cell Signaling | 5174 |
| β-tubulin | rabbit pAb | Santa Cruz | Sc-9104 |
