# Supplementary material for: VDAC1 negatively regulates melanogenesis through the Ca2+-calcineurin-CRTC1-MITF pathway
Source: Life Sci Alliance. 2022 Jun 1;5(10):e202101350. doi: 10.26508/lsa.202101350 (PMC9160443; doi:10.26508/lsa.202101350)
Supplement: Supplementary file 3 [file LSA-2021-01350_TableS3.docx]

**Supplementary Tables**

**Table S3.** Primer details for gene amplifications.

| Genes | | | Primer sequences |
| --- | --- | --- | --- |
| *VDAC1* | mouse | F | CTCCCACATACGCCGATCTT |
|  |  | R | GCCGTAGCCCTTGGTGAAG |
|  | human | F | GCCGAGAGGACGAACTCCAG |
|  |  | R | TGTTCGGCGAGAATGACGAA |
| *TYR* | mouse | F | CGTAATCCTGGAAACCATGACA |
|  |  | R | GTCAAACTCAGACAAAATTCCACATC |
|  | human | F | CTTGTGAGCTTGCTGTGTCG |
|  |  | R | GTGAGGTCAGGCTTTTTGGC |
| *TYRP1* | mouse | F | GATCCGTTCTAGAAGCACCAAGA |
|  |  | R | CCTCAGCATAGCGTTGATAGTGA |
|  | human | F | ATGTCGCTCAGTGCTTGGAA |
|  |  | R | GACTTCGAACAGCAGGGTCA |
| *TYRP2* | mouse | F | TAATTGTGGAGGCTGCAAGTTC |
|  |  | R | AGGATGGCCGGCTTCTTC |
|  | human | F | AACTCCCTTCCCTGCATGTG |
|  |  | R | TTGTGACCATAGGGGCCAG |
| *MITF* | mouse | F | AGATTTGAGATGCTCATCCCC |
|  |  | R | GATGCGTGATGTCATACTGGA |
|  | human | F | ACCTTCTCTTTGCCAGTCCA |
|  |  | R | TTGGGCTTGCTGTATGTGGT |
| *GAPDH* | mouse | F | AGGTCGGTGTGAACGGATTTG |
|  |  | R | GGGGTCGTTGATGGCAACA |
|  | human | F | TGACTTCAACAGCGACACCCA |
|  |  | R | CACCCTGTTGCTGTAGCCAAA |
