# Supplementary material for: VDAC1 negatively regulates melanogenesis through the Ca2+-calcineurin-CRTC1-MITF pathway
Source: Life Sci Alliance. 2022 Jun 1;5(10):e202101350. doi: 10.26508/lsa.202101350 (PMC9160443; doi:10.26508/lsa.202101350)
Supplement: Supplementary file 4 [file LSA-2021-01350_TableS4.docx]

**Supplementary Tables**

**Table S4.** Primer details for genotyping of *Vdac1*-knockout mice.

| Primer | sequences |
| --- | --- |
| WT-F | AGGCCCGGCTATGATACCCTTCTT |
| WT-R | GAGCTGGGGACACTTAGTGATTGCT |
| mut-R | ATTGACGTTCTTGCCATCGAGCA |
